# Anterior and posterior surgical approach for vertebral lumbar *Mycobacterium abscessus* osteomyelitis

**DOI:** 10.1093/jscr/rjae521

**Published:** 2024-08-20

**Authors:** Jay Patel, Aldin Malkoc, Muhammad S Ghauri, Luv Amin, Morgan Petersen, Julia Cochrane, Gail Hopkins, Samuel Schwartz

**Affiliations:** Department of Vascular Surgery, California University of Science and Medicine, 1501 Violet Street, Colton, CA 92324, United States; Department of Vascular Surgery, Arrowhead Regional Medical Center, 400 N Pepper Ave, Colton, CA 92324, United States; Department of Vascular Surgery, California University of Science and Medicine, 1501 Violet Street, Colton, CA 92324, United States; Department of Neurosurgery, California University of Science and Medicine, 1501 Violet St, CA 92324, United States; Department of Vascular Surgery, California University of Science and Medicine, 1501 Violet Street, Colton, CA 92324, United States; Department of Vascular Surgery, California University of Science and Medicine, 1501 Violet Street, Colton, CA 92324, United States; Department of Vascular Surgery, California University of Science and Medicine, 1501 Violet Street, Colton, CA 92324, United States; Department of Vascular Surgery, Arrowhead Regional Medical Center, 400 N Pepper Ave, Colton, CA 92324, United States; Department of Orthopedic Surgery, Arrowhead Regional Medical Center, 400 N Pepper Ave, CA 92324, United States; Department of Vascular Surgery, Arrowhead Regional Medical Center, 400 N Pepper Ave, Colton, CA 92324, United States

**Keywords:** vertebral osteomyelitis, thoracoabdominal incision, Mycobacterium abscessus, anterior lumbar interbody fusion

## Abstract

*Mycobacterium abscessus* (*M. abscessus*) infections primarily affect immunocompromised patients who commonly present with non-orthopedic infections. We present a case of a 63-year-old female presented with persistent back pain and radicular pain. Computed tomography and magnetic resonance imaging showed a large multiloculated anterior epidural abscess. We show here the unique occurrence of lumbar *M. abscessus* vertebral osteomyelitis, which was treated with L2 and L3 corpectomies, anterior lumbar interbody fusion, and posterior instrumentation via an anterolateral thoracoabdominal (TA) incision. Vascular surgery provided L1–L4 spine exposure via a left anterolateral TA incision, whereas orthopedic surgery performed L2 and L3 corpectomies with lumbar cage placement and posterior instrumentation in two separate procedures. The patient was discharged to a skilled nursing facility, retaining all neurological function, and is progressing well on follow-up.

## Introduction

*Mycobacterium abscessus* (*M. abscessus*) infections primarily affect immunocompromised patients commonly presenting with pulmonary, skin, and soft tissue infections [1]. Vertebral osteomyelitis (VO) caused by *M. abscessus* is rare and often requires surgical intervention for symptoms of spinal compression or instability [2]. The anterior approach is commonly utilized in cases resembling Pott’s disease, offers direct access to pathology, and facilitates fusion with minimal muscle dissection. However, few cases utilize this approach for *M. abscessus*-related osteomyelitis after failed medical therapy [3, 4].

## Case report

A 63-year-old female with a medical history of lumbar VO, bilateral psoas abscesses, and epidural abscess presented to the emergency department with lower back pain radiating to the bilateral lower extremities. The patient was treated at an outside hospital 1 year prior to surgery with antibiotics and bilateral interventional radiology (IR) drain placement for psoas abscesses with cultures positive for *M. abscessus*. The patient declined surgical intervention for nerve root compression. The patient did not show clinical improvement over the following months, and imaging showed disease progression. On presentation, the patient had spinal tenderness on palpation and radicular low back pain, without compromising autonomic function. Her right psoas drain contained purulent material.

**Figure 1:**
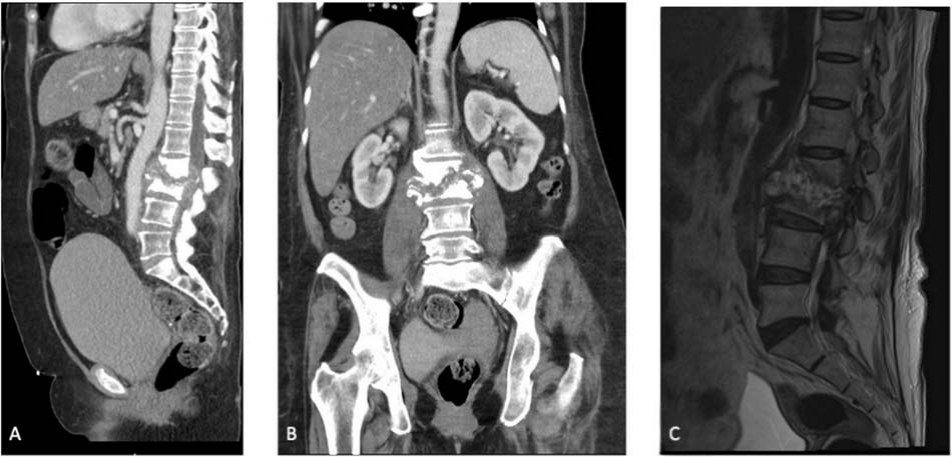
(A) Sagittal section of a CT scan depicting L2–L3 discitis-osteomyelitis. (B) Coronal section of a CT scan depicting L2–L3 discitis-osteomyelitis. (C) Sagittal MRI of the lumbar spine demonstrating severe L2–L3 discitis and L2–L3 osteomyelitis with a large anterior epidural abscess causing severe spinal stenosis at the L2 and L3 levels.

Initial laboratory results showed a normal white blood cell count of 9.3 × 10^3^/μL, elevated erythrocyte sedimentation rate of 47 mm/h, and high-sensitivity C-reactive protein of 8.26 mg/dL. Preoperative computed tomography (CT) and magnetic resonance imaging (MRI) of the patient’s abdomen and pelvis demonstrated L2–L3 discitis-osteomyelitis with a large anterior epidural abscess (Fig. 1A–C).

**Figure 2:**
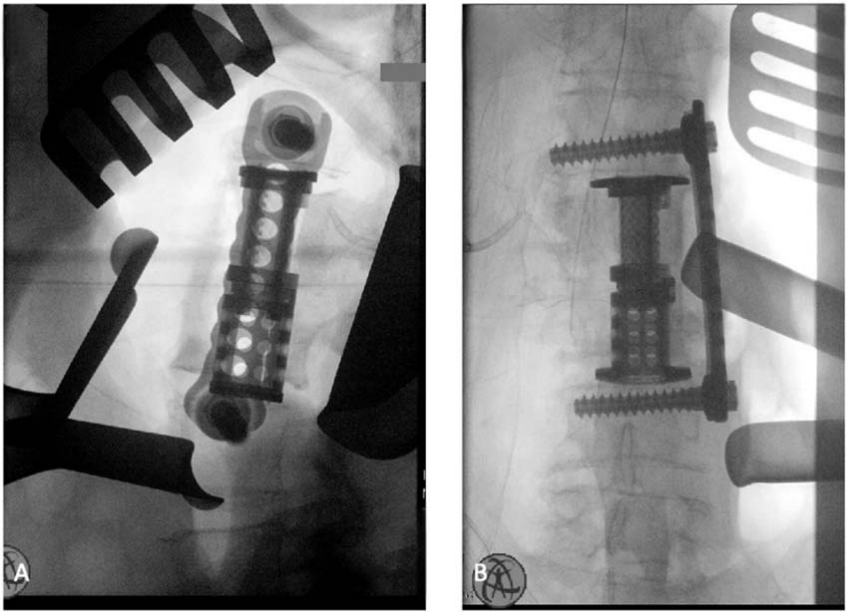
(A) Anterior intraoperative fluoroscopy demonstrating final titanium cage placement spanning from L1 to L4. (B) Lateral intraoperative fluoroscopy demonstrating final titanium cage placement spanning from L1 to L4.

**Figure 3:**
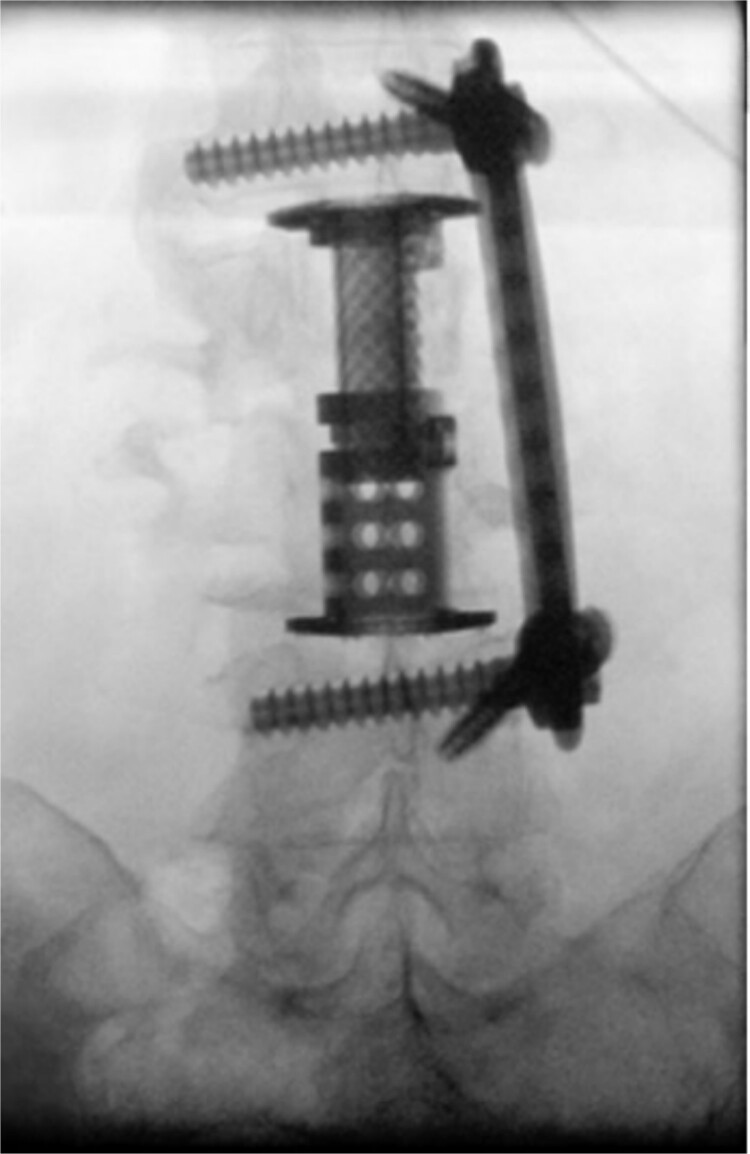
Intraoperative fluoroscopy depicting two pedicle screws posteriorly placed in a percutaneous fashion with a 5.5 × 100 mm^2^ rod placed in the pedicle tulips.

The patient was placed in the operating room for anterior lumbar interbody fusion via thoracoabdominal (TA) incision for retroperitoneal exposure. The patient was placed in the right-lateral decubitus position. The 10th rib interspace was identified, and a curvilinear incision was made from the midaxillary line and extended anteriorly and inferiorly toward the umbilicus. The retroperitoneal plane was entered, and the 11th rib was cut and morcellated for lumbar cage placement. The diaphragm was divided radially with electrocautery to further expose the abdominal cavity and retroperitoneal space. The lateral border of the psoas muscle was exposed and dissected into the anterior vertebrae L1–L4. The aorta and abdominal viscera were retracted toward the patient’s right side using an Omni retractor. Copious necrotic tissue and caseating granulomas were removed. The L2–L3 vertebrae were exposed, corpectomy was performed, and a titanium cage was placed with a plate spanning from L1 to L4 (Fig. 2A and B). To achieve added stability, a second surgery for posterior percutaneous screw placement without lumbar fusion was performed (Fig. 3). The patient recovered without complications and was subsequently discharged to a skilled nursing facility, and ultimately home. At the 1-month follow-up visit, the patient’s TA incision had completely healed, and there was no evidence of recurrent infection.

## Discussion

*Mycobacterium abscessus* predominantly affects immunocompromised individuals, infecting pulmonary, skin, and other soft tissues [1]. VO due to *M. abscessus* is very rare, with few cases reported in the literature. The initial pharmacological management typically involves antibiotic therapy; however, some patients may fail to respond or develop complications. The sequelae of spinal infection include bone destruction and soft tissue damage, which can lead to vertebral instability, spinal cord compression, and neurological deficits. We present the case of a patient who failed to improve with conservative pharmacological management that required surgical intervention.

Currently, surgical management for VO is reserved for chronic disease, as seen in our patient, and evidence of spinal cord compression, focal neurological deficit, or disseminated infection leading to sepsis [5]. Despite this, there is no consensus in the medical literature that delineates the optimal surgical approach for VO. The surgical approach depends on the location of infection within the vertebral column. In a previous case report, Moral et al. described that a similar two-staged anterior and posterior approach were utilized [6]. Additionally, in another case study by Kato et al., a right retroperitoneal approach was used in a single staged procedure [7]. However, our case was performed with an anterolateral TA for retroperitoneal exposure and a subsequent posterior approach. The anterior approach allows direct access to multiple vertebral levels at the pathology site for optimal decompression and tissue debridement.

The severity of the infection and prolonged attempts at medical management led to a difficult surgical case. Months of admission and courses of antibiotics failed to reduce the infection, necessitating surgical management. The VO extended through multiple vertebral bodies and was complicated by the presence of a spinal epidural abscess, which contraindicated an initial posterior approach. The breadth and level of vertebral involvement made a purely anterior approach nonviable given that imaging showed VO from L2 to L4. A multidisciplinary surgical team consisting of vascular and orthopedic surgeries was necessary for exposure. In addition to surgical collaboration, this case required close involvement of infectious disease consultation for inpatient and outpatient antibiotic treatment optimization. There are no current guidelines that recommend a particular surgical approach. Currently, an anterior approach, posterior approach, or combined approach are the most heavily utilized surgical approaches for the treatment of pyogenic VO [8]. The approach is dependent upon the surgical goals and needs for additional stabilization [9, 10]. However, the combined approach has been shown to decrease rates of infection and revision surgery [8]. National Jewish health was consulted for expertise in treating nontuberculous mycobacterial infections, given the rarity and resistance of *M. abscessus.*

Surgical management facilitated intraoperative cultures and susceptibility testing, and the patient was discharged on omadacycline, linezolid, and imipenem managed by outpatient infectious disease. According to Novosad et al., the most commonly used antibiotics in *M. abscessus* infections consist of amikacin, macrolides, and imipenem [11]. However, due to multidrug resistance and differing antibiotic sensitivities between patients, there is no ideal recommended drug combination [12]. At a 1-month follow-up, the patient continued to demonstrate intact neurological function and functional status, including the ability to ambulate and perform activities of daily living. Given the complex disease presentation and significant risk of neurological compromise, our interdisciplinary anterolateral surgical approach proved to be successful. Future studies are warranted to validate our multidisciplinary surgical approach and better understand how to optimize the surgical management of VO.

## Conflict of interest statement

All authors have nothing to disclose or any conflicts of interest. 

## Funding

This project received no funding.
